# Pre‐diagnostic manifestations of Alzheimer’s disease: A systematic review and meta‐analysis of longitudinal studies

**DOI:** 10.1002/alz.085949

**Published:** 2025-01-03

**Authors:** Sedigheh Zabihi, Rosario Isabel Espinoza Jeraldo, Rifah Anjum, Christine Carter, Moïse Roche, Jonathan P Bestwick, Sarah Morgan‐Trimmer, Claudia Cooper, Charles R Marshall, Yvonne Birks, Mark Wilberforce, Fiona M Walter

**Affiliations:** ^1^ Queen Mary University of London, London United Kingdom; ^2^ University of Exeter, Exeter United Kingdom; ^3^ University of York, York United Kingdom

## Abstract

**Background:**

Alzheimer’s disease (AD) is associated with a range of non‐cognitive symptoms that can be early or even presenting features. Better recognition of pre‐diagnostic symptoms of AD would support improved early detection and diagnosis.

**Method:**

To identify possible prodromal symptoms of AD, we systematically searched three electronic databases for prospective longitudinal studies to March 2023, that reported the risk of AD diagnosis associated with non‐cognitive symptoms. Meta‐analyses were performed to assess the associations between a commonly reported symptom and AD. We estimated pooled odds ratios (OR) using random‐effects model.

**Result:**

Thirty studies were included with the majority focused on neuropsychiatric symptoms. Quantitative analysis of fourteen studies showed depression to be associated with subsequent AD diagnosis (pooled OR = 1.80; 95% CI: 1.29 to 2.50; I^2^ = 95.8%) and individual reports of the association showed it to be stronger later in life and closer to AD diagnosis. In contrast with the literature, the evidence on the association between anxiety, sleep difficulties, personality changes and psychotic symptoms, and incident AD was inconsistent.

We found evidence that autonomic symptoms including urinary dysfunction, constipation, fatigue, syncope and collapse might be pre‐diagnostic symptoms of AD recorded up to 10 years before receiving an AD diagnosis, and there was strong evidence of an association between hypotension and incident AD (pooled OR = 1.94; 95% CI: 1.88 to 2.01; I^2^ = 0.0%). We were also able find evidence for the association between weight loss and subsequent AD diagnosis within 10 years (pooled OR = 1.46; 95% CI: 1.22 to 1.74; I^2^ = 57.5%). Furthermore, the quantitative synthesis of the evidence on hearing loss (pooled OR = 1.47; 95% CI: 1.14 to 1.89; I^2^ = 87.6%) and spondylosis (pooled OR = 1.55; 95% CI: 1.34 to 1.80; I^2^ = 68.2%) showed both to be associated with AD diagnosis up to 10 years after identification of these sensory symptoms.

**Conclusion:**

Our findings suggest that improved recognition of early non‐cognitive presentations is key in facilitating access to timely diagnosis of AD, especially as the emergence of disease‐modifying therapies for AD increases the importance of early detection.

